# Skeletal Site-Specific Lipid Profile and Hematopoietic Progenitors of Bone Marrow Adipose Tissue in Patients Undergoing Primary Hip Arthroplasty

**DOI:** 10.3390/metabo15010016

**Published:** 2025-01-04

**Authors:** Drenka Trivanović, Marko Vujačić, Aleksandra Arsić, Tamara Kukolj, Milica Rajković, Nikola Bogosavljević, Zoran Baščarević, Mirjana Maljković Ružičić, Jovana Kovačević, Aleksandra Jauković

**Affiliations:** 1 Group for Hematology and Stem Cells, Institute for Medical Research, National Institute of Republic of Serbia, University of Belgrade, 11000 Belgrade, Serbia; tamara.kukolj@imi.bg.ac.rs (T.K.); milica.rajkovic@imi.bg.ac.rs (M.R.); aleksandra@imi.bg.ac.rs (A.J.); 2 Institute for Orthopedy Banjica, 11000 Belgrade, Serbia; marko.vujacic@iohbb.edu.rs (M.V.); boga19@gmail.com (N.B.); zoran.bascarevic@iohbb.edu.rs (Z.B.); 3 Faculty of Medicine, University of Belgrade, 11000 Belgrade, Serbia; 4 Centre of Research Excellence in Nutrition and Metabolism, Institute for Medical Research, National Institute of Republic of Serbia, University of Belgrade, 11000 Belgrade, Serbia; aleksandra.arsic@imi.bg.ac.rs; 5 Faculty of Mathematics, University of Belgrade, 11000 Belgrade, Serbia; mirjana.maljkovic.ruzicic@matf.bg.ac.rs (M.M.R.); jovana.kovacevic@matf.bg.ac.rs (J.K.); 6 Institute for Artificial Intelligence Research and Development of Serbia, 21000 Novi Sad, Serbia

**Keywords:** bone marrow adipose tissue, osteoarthritis, fatty acid, stem cells, hematopoietic progenitors

## Abstract

Background/Objectives: Bone marrow adipose tissue (BMAT) has been described as an important biomechanic and lipotoxic factor with negative impacts on skeletal and hematopoietic system regeneration. BMAT undergoes metabolic and cellular adaptations with age and disease, being a source of potential biomarkers. However, there is no evidence on the lipid profile and cellularity at different skeletal locations in osteoarthritis patients undergoing primary hip arthroplasty. Methods: Acetabular and femoral bone marrow (BM) and gluteofemoral subcutaneous adipose tissue (gfSAT) were obtained from matched patients undergoing hip replacement surgery. BM, BMAT, and gfSAT were explored at the levels of total lipids, fatty acids, and cells by using thin-layerand gas chromatography, ex vivo cellular assays, and flow cytometry. Results: BMAT content was significantly higher in femoral than in acetabular BM. Total lipid analyses revealed significantly lower triglyceride content in femoral than in acetabular BMAT and gfSAT. Frequencies of saturated palmitic, myristic, and stearic acids were higher in femoral than in acetabular BMAT and gfSAT. The content of CD45^+^CD34^+^ cells within femoral BMAT was higher than in acetabular BMAT or gfSAT. This was associated with a higher incidence of total clonogenic hematopoietic progenitors and late erythroid colonies CFU-E in femoral BMAT when compared to acetabular BMAT, similar to their BM counterparts. Conclusions: Collectively, our results indicate that the lipid profiles of hip bone and femoral BMAT impose significantly different microenvironments and distributions of cells with hematopoietic potential. These findings might bring forth new inputs for defining BMAT biology and setting novel directions in OA disease investigations.

## 1. Introduction

Bone marrow adipose tissue (BMAT) is an important constituent of bone and bone marrow (BM) microenvironments and can represent over 10% of total adipose tissue mass in adults. BM adipocytes are functionally distinguishable from extra-medullary adipocytes, and BMAT still represents a great challenge to be assessed. BMAT is involved in the regulation of the functional relationships with bone and blood cell production [1,2,3]. The expansion of BMAT occurs during physiological aging and can be upregulated in distinct settings such as anorexia, obesity, osteoporosis, hyperlipidemia, estrogen deficiency, and treatment with pharmacotherapies such as glucocorticoids and thiazolidinediones [4]. During aging, BMAT expansion and related lipotoxicity are potent contributors of bone loss and dysregulated hematopoiesis [5,6,7]. However, the majority of evidence comes from rodent studies.

The impacts of BMAT on bone health and hematopoiesis are still not fully clarified. It has been shown that hematopoietic stem cells (HSCs) and short-term progenitors are reduced in frequency in the adipocyte-rich vertebrae in mice [8]. Moreover, committed adipogenic cells residing in bone inhibit hematopoiesis and femoral bone healing and regeneration [9]. On the other side, it has been described that BMAT secretes the stem cell factor (SCF) involved in hematopoiesis regulation both at the steady state and upon stress in mice [10]. Importantly, the effect of SCF derived from bone marrow adipocytes on hematopoietic cells depends on skeletal sites. Namely, SCF deletion using Adipoq-Cre/ER doesn’t impact HSC frequency in long bones with relatively low BMAT content but induces HSC depletion in tail vertebrae with high BMAT content [11]. Therefore, the crosstalk of BMAT and hematopoietic cells depends on the skeletal site. Marrow adipocytes can be considered local sources of energy in the form of fatty acids (FAs). Recent studies showed the dependence of emergency hematopoiesis (proliferation of hematopoietic progenitor proliferation and myeloid cells) [12] and clonal hematopoiesis [13] on FAs released from BMAT in mice.

However, data on the association between BMAT and hematopoietic cells in humans are still lacking, particularly in the context of different skeletal sites. Hip joint osteoarthritis (OA) is an incurable disease affecting articular cartilage, periarticular and subchondral bone, the capsule, synovial membrane, fluid, and calcified cartilage [14]. Femoro-acetabular impingement induces structural damage and stress response, leading to sclerotic and lytic bone lesions and the formation of BM lesions [15]. The maintenance of acetabular (subchondral) bone health appears to be important for acetabular cup stability during primary and revision hip arthroplasty [16]. BM specimens obtained from patients undergoing hip replacement represent a frequently used source of healthy control counterparts for the investigation of various non-malignant and malignant states of musculoskeletal and hematopoietic systems [15]. Thus, it is of great importance to define the characteristics of BMAT and associated progenitor cells in acetabular and femoral BM specimens.

Here, we performed a cross-sectional study to characterize lipids and hematopoietic progenitors in hip (acetabular) and femoral BMAT in osteoarthritis patients. For the first time, we provided evidence on lipid profile and hematopoietic cells associated with human BMAT, contributing to a better understanding of human BMAT biology at distinct skeletal sites in OA patients.

## 2. Materials and Methods

### 2.1. Human Subjects

Patient-matched acetabular and femoral metaphyseal BM and gluteofemoral subcutaneous adipose tissue (gfSAT) specimens were obtained from patients undergoing total hip arthroplasty. In total, independent specimens from 11 patients (6 women, 5 men, mean age 66 ± 11 years (from 48 to 86 years), body mass index (BMI) 27.9 ± 4.4 kg/m^2^ (from 22.48 to 37.55 kg/m^2^)) were included. Patients who experienced hip pain, restricted function and disability, and were unresponsive to pharmacologic treatments were included in the study. Patients with fractures, malignancies, metabolic diseases, or infections were excluded from the study. Samples were collected at the Institute for Orthopedy Banjica, Belgrade, and analyzed in a patient-matched manner. The study was performed according to the Declaration of Helsinki. All participants signed informed consent prior to participation in the study. Samples were processed within 24 h after surgery and were received in the laboratory.

### 2.2. Processing of BM Samples and Preparation of BMAT

Before processing, femoral BM was minced by using forceps and scalpels to obtain morphologically similar specimens to acetabular BM (reaming material). BM samples were weighed and washed with phosphate-buffered saline (PBS, Capricorn Scientific, Ebsdorfergrund, Germany) to collect cells. The suspension was drained through cell strainers with a 100 µm pore size (Stem Cell Technologies, Vancouver, BC, Canada). BM mononuclear cells were isolated using a Ficoll density gradient (1.077 ± 0.001 g/cm^3^, Capricorn Scientific, Germany). Bone marrow mononuclear cells were re-suspended in MEM—Minimum Essential Media (Capricorn Scientific, Germany)—supplemented with 1% penicillin/streptomycin (Capricorn Scientific, Germany), 100 mM of Hepes (PAN-BioTech, Aidenbach, Germany), and 10% of fetal calf serum (FCS, Capricorn Scientific, Germany), assigned as standard growth medium (SGM). Fresh mononuclear cell counts and viability were scored by counting in a Neubauer chamber in Trypan blue or Tuerk solution.

The floating fatty layer (BMAT) was collected after each BM washing step until Ficoll separation by using a plastic Pasteur pipette, and it was kept in a polypropylene tube at room temperature. Collected BMAT was washed 2 times with pre-warmed Krebs Ringer Buffer (Merck, Darmstadt, Germany) supplemented with 1% penicillin/streptomycin, 100 mM Hepes, and 0.5% w/v FA-free bovine serum albumin (BSA, Capricorn Scientific, Germany) (KRBS). The tissue was washed by 2 centrifugations at 300× *g* for 5 min. The aforementioned steps were performed in accordance with established protocol [17]. The resulting volume of BMAT (both liquid and adipocyte aggregate fractions) was estimated by using a pipette. After extraction, one part of the BMAT samples was used for enzymatic digestion (Section 2.5) and the other part was kept at −80 °C for lipid analysis (Section 2.6).

### 2.3. Processing of Subcutaneous gfSAT

After obtaining the tissue in sterile condition, gfSAT was placed into a sterile Petri dish, where all non-fatty contents (muscles, tendons, etc.) were removed by a scalpel and gfSAT was cut into small pieces by using forceps and scalpels. gfSAT was weighed and washed 2 times in KRBS to remove residual blood.

### 2.4. Fluorescent Visualization of Ex Vivo Adipose Tissues

Pieces of BMAT and gfSAT obtained during the first washing steps of BM specimens (described in Section 2.2 and Section 2.3) were fixed in 4% paraformaldehyde (Superlab, Belgrade, Serbia) for 1 h. Thin layers of tissue were made by using a scalpel and tissues were permeabilized with 0.1% Triton-X100 (Sigma Aldrich, Burlington, MA, USA) in PBS for 10–15 min at room temperature. Phalloidin (Sigma Aldrich Biochemie, Hamburg, Germany), BODIPY™ 493/503 (Invitrogen, Waltham, MA, USA), and DAPI (Santa Cruz Biotechnology, Santa Cruz, CA, USA) were used for the staining of F-actin, neutral lipids, and nuclei, respectively. Tissue layers were mounted with a drop of Fluoromount™ Aqueous Mounting Medium (Sigma Aldrich, Burlington, MA, USA) onto microscopy slides. Fluorescence was checked by epifluorescence microscopy (Olympus, Tokio, Japan).

### 2.5. Isolation of Cells Associated with BMAT and gfSAT

For enzyme digestion, BMAT and gfSAT were incubated in a pre-warmed collagenase II (Stem Cell Technologies, Canada) solution (10 U/mL). A total of 0.5 mL of collagenase supplemented with 500 µM CaCl_2_ (Sigma Aldrich) was added per each mL of the BMAT or gfSAT. Tissues were digested for 1 h in humidified conditions at 37 °C, and tubes were mixed by gentle agitation every 10 min. At the end of the digestion, BMAT and gfSAT obtained a smooth viscose liquid appearance and were drained through a cell strainer (300 µm, PluriSelect, Leipzig, Germany) to remove cellular debris and undigested fragments. The cell strainer was washed 2 times with KRBS and at the end with SGM. After centrifugation at 300× *g* for 5 min at room temperature, stromal vascular fraction cells were pelleted, while adipocyte fractions were left in the floating layers. The lysis of erythrocytes was performed by the incubation of cells in the lysis buffer (0.15 M NH_4_Cl and 10 mM EDTA, AppliChem GmbH, Darmstadt, Germany) for 3 min at room temperature. Fresh cell counts and viability were scored by counting in a Neubauer chamber in Trypan blue (Invitrogen) or Tuerk solution (Superlab).

### 2.6. Lipid Analysis of BMAT: Thin-Layer Chromatography and Gas Chromatography

BMAT samples were homogenized by vortexing and 200 µL was resuspended in 1.5 mL to form a mixture of chlorophorm/methanol (2:1) (Superlab, Serbia). After centrifugation, the organic layer evaporated to dryness and was resuspended in a chloroform–methanol mixture. Thin-layer chromatography (TLC) with silica gel as the adsorbent was used to evaluate qualitative changes in the lipid’s classes of BMAT. For gas chromatography (GC) fatty acid analysis, 200 µL of lipid extract was directly trans-esterified with 3 N HCl in methanol (Superlab, Serbia) at 85 °C for 60 min, as previously described [18]. FA methyl esters were analyzed using a gas chromatograph SHIMADZU 2014 (Kyoto, Japan), equipped with a capillary column RESTEK Rtx 2330 (RESTEK, Bellefonte, PA, USA). The temperature program was 140–210 °C for 3°/min. Individual FAs were identified by comparison with the retention time of FA methyl esters commercial standards PUFA-2 (Supelco, Inc., Bellefonte, PA, USA). The results are presented as a percentage of the total FAs.

### 2.7. Assessment of Hematopoietic Progenitor Growth In Vitro

A single cell suspension of mononuclear or nucleated cells obtained in Section 2.2 and Section 2.5, respectively, was seeded in human HSC003 methylcellulose complete media (R&D systems, Minneapolis, MN, USA) containing 25% FCS, 2% BSA, 2 mM L-Glutamine, 2-mercaptoethanol 5 × 10^−5^ M, rh SCF 50 ng/mL, rh granulocyte–macrophage colony-stimulating factor 10 ng/mL, rh IL-3 10 ng/mL, and rh erythropoietin 3 IU/mL. Cells were seeded in 3 technical replicates in 24-well plates at a concentration of 3.5 × 10^4^ cells/well and grown in standard conditions (37 °C, 5% CO_2_ in humidified atmosphere). After 7 days, the late-stage CFU-erythroid (CFU-E) colonies were scored, while the early-stage burst forming unit erythroid (BFU-E) and granulocyte–monocyte/macrophage (CFU-GM) and multilineage colonies of granulocytes, erythrocytes, macrophages, and megakaryocytes (CFU-GEMM) were scored after 14 days.

### 2.8. Flow Cytometry

To determine the presence of hematopoietic cells in isolated cell populations, surface marker expression was analyzed by flow cytometry. Cells were washed with PBS supplemented with 5% FCS (FACS buffer) and centrifuged at 400× *g* for 5 min. Incubation with anti-human fluorochrome-conjugated antibodies was performed according to the manufacturer’s recommendations in FACS buffer in a volume of 100 µL at 4 °C for 45 min in the dark. Afterwards, cells were washed with FACS buffer and fixed with 4% paraformaldehyde for further analyses. Multicolor flow cytometry was performed using FACS Lyrics (Becton Dickinson, Franklin Lakes, NJ, USA). All data analyses were performed using FlowJo V10 software (TreeStar, Ashland, OR, USA).

### 2.9. Data Analysis and Statistics

Normal distribution was assessed using the D’Agostino and Pearson normality test where the sample size allowed. The number of investigated individual donors (biological replicates) is indicated as *n* for each experiment. The statistical significance was determined by non-parametric tests and a post hoc test for multiple comparisons. Bars represent means ± SD. For comparison between BM, BMAT, and gfSAT, the Kruskal–Wallis test was used following proper post hoc tests. * *p* < 0.05 indicates a significant difference. All statistical analyses were performed using GraphPad Prism 8.0.1 software (San Diego, La Jolla, California, USA). The data were preprocessed by calculating Z-scores by using Python library SciPy prior to the statistical analyses. Clustered heatmap and linear discriminant analysis were performed using the Python libraries scikit-learn and seaborn.

## 3. Results

### 3.1. Human Bone Marrow Adipose Tissue

**Figure 1:**
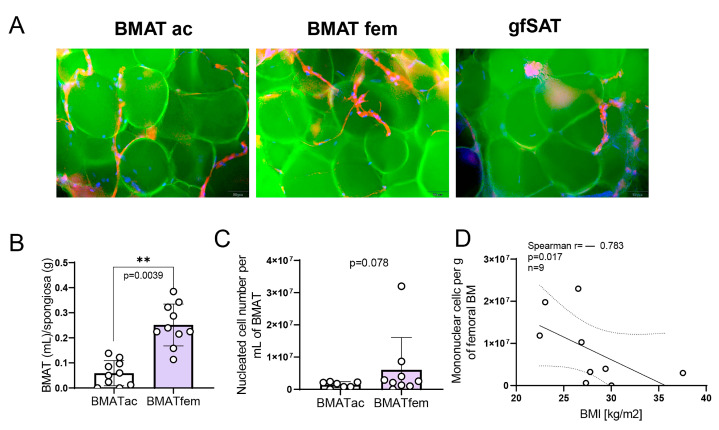
**Human bone marrow adipose tissue (BMAT) and the importance of skeletal sites and BMI**. (**A**) Fluorescent staining of neutral lipids (green), F-actin (red), and the nucleus (blue). Representative images are presented. Scale bar is 10 µm. (**B**) BMAT content in acetabular and femoral bone marrow. (**C**) Number of nucleated cells per mL of BMAT counted in BMAT ac and BMAT fem isolated after collagenase digestion. (**D**) Linear regression with the correlation coefficients of patient BMIs and the distribution of bone marrow mononuclear cells. Non-normally distributed values were analyzed with the Mann–Whitney test, where ** *p* < 0.01. Results are shown as mean ± SD.

To assess the identity of the isolated adipose tissues, we stained fresh specimens to visualize adipocyte components, namely the lipid droplet (neutral lipid), cytoskeleton (phalloidin-positive F-actin fibers), and compressed nuclei. We did not observe permanent differences when comparing acetabular BMAT (BMAT ac), femoral BMAT (BMAT fem), and gfSAT (Figure 1A). We observed significantly higher BMAT content in femoral than in acetabular BMAT (Figure 1B). In addition, the number of nucleated cells per mL of BMAT was slightly higher in BMAT fem than in BMAT ac, although without statistical significance (Figure 1C). Interestingly, the number of mononuclear cells in the femoral BM decreased with the BMI of patients (Figure 1D), suggesting a high BMI as a potential indicator of femoral cellularity in patients undergoing hip arthroplasty. Altogether, these findings show different BMAT content and the number of BMAT-associated cells from distinct skeletal sites in patients undergoing hip arthroplasty.

### 3.2. Lipid Content and Fatty Acids in Human Bone Marrow Adipose Tissue

**Figure 2:**
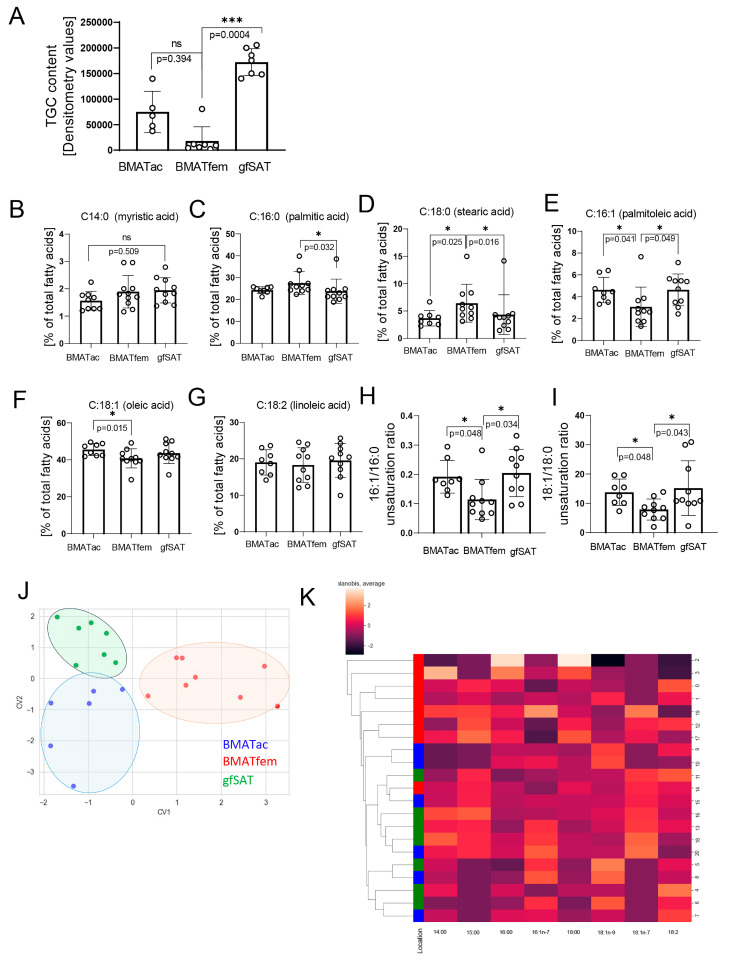
**Exploratory lipid content and fatty acid profile analysis.** Individual FA proportion in adipose tissues isolated from acetabulum, femur, and gfSAT (n = 10). (**A**) Triglyceride content determined by thin-layer chromatography. (**B**–**G**) FA ratios were calculated from proportion of total fatty acids. (**H**,**I**) Unsaturation ratios of 16:0/16:1 and 18:0/18:1 FAs. Non-normally distributed values were analyzed with one-way ANOVA following Dunne’s post hoc test, where * *p* < 0.05; *** *p* < 0.001. Results are shown as mean ± SD. Differentiation of body sites based on fatty acid compositions of adipose tissues using (**J**) linear discriminant analysis and (**K**) clustered heatmap obtained using the Mahalanobis metric and method average.

To further assess the profile of BMAT, we evaluated lipid content and the FA profile in human specimens. Our results indicated the presence of the seven most abundant FAs (Table 1). The results showed a significantly lower content of triglycerides in BMAT ac and gfSAT when compared to BMAT in the femur (Figure 2A). The analyses of FA proportions showed an increased presence of saturated FAs (myristic, palmitic, and stearic) in BMAT fem when compared to BMAT ac and gfSAT (Figure 2B–D). On the other hand, abundances of monounsaturated FAs, palmitoleic and oleic acids, were significantly lower in BMAT fem than BMAT ac (Figure 2E,F), while no differences were observed when linoleic acid (polyunsaturated FA) was analyzed (Figure 2G). Interestingly, unsaturation ratios (16:0/16:1 and 18:0/18:1 FAs) have been shown to be lower in femoral BMAT than BMAT ac and gfSAT (Figure 2H,I). Altogether, our results suggest differences between femoral BMAT on one side and acetabular BMAT and gfSAT on the other (Figure 2J,K) in terms of the FA profile in osteoarthritis patients. Based on this, femoral BMAT can be delineated from other adipose tissues.

### 3.3. Associations of Fatty Acid Distribution and Patient Data

**Figure 3:**
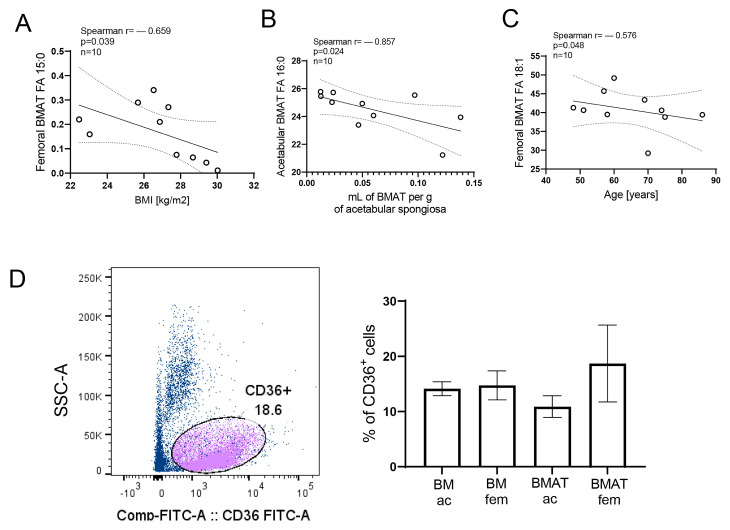
**Association of FA distribution in BMAT and patient data.** (**A**–**C**) Linear regression with Spearman correlation coefficients of patient data and FA distribution in BMAT. (**D**) Gating strategy and percentages of CD36+ cells (violet subset within total cell/singlets (dark blue). Non-normally distributed values were analyzed with one-way ANOVA following Dunne’s post hoc test. Results are shown as mean ± SD.

After observing site-specific differences in the adipose tissue of patients undergoing hip arthroplasty, we estimated correlations between FAs and the demographic data of patients and other parameters. Palmitic and oleic acids were shown to be most present FAs in BMAT ac, BMAT fem, and gfSAT. Our results showed a decrease in essential FAs, although there was a minor amount of pentadecanoic acid (Figure 3A) in BMAT fem related to BMI. The presence of palmitic acid 16:0 in BMAT ac decreased with BMAT content at this skeletal site (Figure 3B). In addition, the frequency of 18:1n-9 (oleic acid) in BMAT fem declined with age of patients (Figure 3C). CD36 is a high-affinity receptor for long-chain FAs that has been shown to facilitate net FA uptake into human adipocytes and regulate lipid metabolism [19]. As we observed different patterns of triglycerides and FAs in BMAT and gfSAT, we assumed that the expression of CD36 might be associated with shown differences. However, we could not find significant differences in CD36 expression in cells within BM and BMAT (Figure 3D).

### 3.4. Skeletal Site Governs Distribution of Hematopoietic Progenitors in BMAT

**Figure 4:**
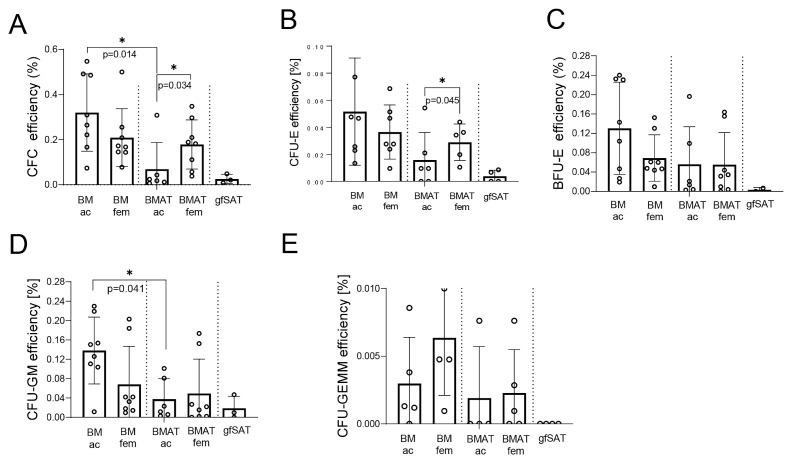
**Committed hematopoietic progenitors distributed in BMAT from different skeletal sites.** Presence of committed hematopoietic progenitors represented as (**A**) total CFC; (**B**) CFU-E; (**C**) BFU-E; (**D**) CFU-GM; and (**E**) CFU-GEMM. Non-normally distributed values were analyzed with one-way ANOVA following Dunne’s post hoc test, where * *p* < 0.05. Results are shown as mean ± SD.

**Figure 5:**
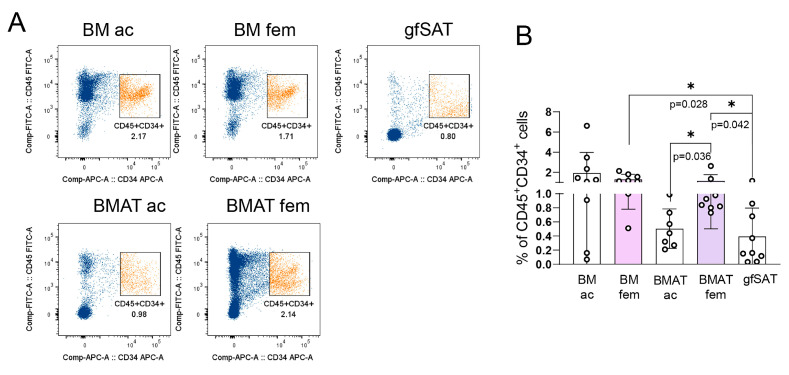
**CD45^+^ CD34^+^ cells in BMAT from different skeletal sites.** Presence of CD45^+^ CD34^+^ cells in nucleated cells of bone marrow, BMAT, and gfSAT. (**A**) Gating strategy and representative dot plots (orange subset represents CD45^+^CD34^+^ population within total cell/singlets (dark blue); (**B**) percentage of CD45^+^ CD34^+^ cells. Non-normally distributed values were analyzed with one-way ANOVA following Dunne’s post hoc test, where * *p* < 0.05. Results are shown as mean ± SD.

In addition to the lipid profile of BMAT, we explored the presence of cells with hematopoietic progenitor activity and properties. Clonogenic assays showed a significantly lower frequency of total committed progenitors within BMAT ac compared to the acetabular BM counterpart as well as BMAT fem (Figure 4A). Considering the late and early erythroid progenitor colonies (CFU-E and BFU-E), we observed a significantly higher content of CFU-E (direct precursor of mature erythrocytes) in BMAT fem when compared to BMAT ac, while our analysis of BFU-Es did not show significant differences (Figure 4B,C). On the other hand, the presence of CFU-GMs was significantly lower in BMAT ac compared to the corresponding BM counterpart. We did not observe differences in CFU-GEMMs, and this is probably the consequence of the low incidence of these progenitors coupled with great inter-patient variability (Figure 4E). As it might be expected, the presence of hematopoietic progenitors within the stromal vascular fraction of gfSAT was negligible. In addition, we analyzed the expression of CD45 and CD34 as markers of hematopoietic progenitor cells (Supplementary Materials). Our results showed that the distribution of CD45^+^CD34^+^ cells was significantly higher in femoral than in acetabular BMAT and gfSAT. The content of CD45^+^CD34^+^ cells within gfSAT was significantly lower than in femoral BM and BMAT (Figure 5A,B). Interestingly, we did not observe a significant difference in CD45^+^CD34^+^ cell presence between femoral BM and BMAT counterparts. Taken together, our results showed that femoral BMAT represents an almost equal microenvironment for committed hematopoietic progenitors as femoral BM. This was not the case with acetabular BMAT, where almost all CFUs and CD45^+^CD34^+^ cells were present at a lower extent than in acetabular BM.

## 4. Discussion

In this cross-sectional study, we investigated the BMAT of patients undergoing primary hip arthroplasty due to osteoarthritis (OA). Our findings revealed a distinct lipid profile of human BMAT in acetabular and femoral BMAT coupled with a difference in hematopoietic progenitors at these skeletal sites.

Aging and obesity are important OA risk factors, while the roles of adipose tissue-derived lipids, free FAs, and adipokines on OA progression in weight-bearing joints and bones are not fully elucidated [20,21,22]. However, bone marrow fat, or BMAT, has shown to be implicated in bone marrow adipogenesis and osteoporotic bone disease development [23], reduced bone mineral density and fracture prevalence [24,25], and adverse bone effects in diabetic patients [26].

Our results showed a higher triglyceride content in acetabular BMAT and gfSAT when compared to femoral BMAT. This might indicate a reduced triglyceride metabolism in acetabular BMAT and gfSAT in patients. Namely, a previous study showed lower triglyceride metabolism in osteoporotic versus normal bones, and this was related to the lower osteoblastogenesis and higher osteoclastogenesis in patients who underwent hip arthroplasty due to hip fracture or primary hip OA [27]. Further analyses are needed to emphasize whether triglyceride content is associated with osteogenesis in bone marrow. On the other hand, data on direct triglyceride effects on bone marrow hematopoietic cells are still lacking. It has been described that lowering triglycerides could reduce the risk of aplastic anemia [28]. One can speculate that relatively low triglyceride content in femoral BMAT might explain the increased frequencies of hematopoietic progenitors (and, particularly, late erythroid progenitors) when compared to acetabular BMAT and almost similar progenitor content when compared to the femoral BM counterpart. Thus, our results showed that femoral BMAT represents an almost equal microenvironment for committed hematopoietic progenitors as femoral BM. This was not the case with acetabular bone, where almost all CFUs and CD45^+^CD34^+^ cells were present at a lower extent in BMAT than in BM. Our results are in accordance with a previous report, where it has been found that regulated BM adipocytes (rBMAds) are enriched in saturated fatty acids, providing stronger hematopoietic support than their constitutive BM adipocyte (cBMAd) counterparts [29]. On the other hand, a previous study on rodents showed that in terms of the lipidomic profile, rBMAT is more similar to extramedullary white adipose tissue [30]. This might be explained by the difference in the methodology applied and animal model used.

Our results showed palmitic and oleic acids to be the most present FAs in acetabular and femoral BMAT, as well as gfSAT, which is in line with early findings observed in postmortem human femoral bone fragments [31], bone marrow aspirates from female patients with hematological malignancies [32], and aspirates from hip fracture patients [33]. It is important to mention that the relative content of saturated and unsaturated FAs in BMAT indicates skeletal site-related BMAT composition [34].

Additionally, in terms of FA profiles, our results delineated femoral BMAT from acetabular BMAT and gfSAT in hip OA patients. Thus, the presence of saturated FAs (myristic, palmitic, and stearic) was higher in femoral BMAT when compared to acetabular BMAT and gfSAT. On the other hand, levels of monounsaturated FAs, palmitoleic and oleic acids, were significantly lower in femoral than in acetabular BMAT. Higher intakes of monounsaturated FAs and n-6 polyunsaturated FAs appeared to be associated with an increased risk of bone marrow lesions in healthy middle-aged adults [35]. Thus, it is possible that a lower presence of monounsaturated FAs reflects femoral unaffectedness in hip OA. On the other hand, palmitic acid has shown to be particularly present in femoral BMAT, and this FA achieved negative effects on osteoblasts in vitro [36]. However, further research is necessary to evaluate the osteoblastic potential of femoral cells.

The CD36 molecule (thrombospondin receptor) is a glycoprotein involved in the transport of FAs and lipid metabolism. A previous study showed that CD36 is required for free FA uptake by HSCs during acute bacterial infection and their cell cycle entering to provide a hematopoietic response [37]. Our findings did not show significant differences in CD36 expression by cells from different skeletal sites, suggesting other mechanisms involved in the regulation of FA levels and lipid metabolism.

To relate the demonstrated findings to OA pathology, early OA stages should be included in the study, as well as specimens obtained from healthy age- and sex-matched donors. However, the collection of such material presents ethical concerns. The number of patients recruited in this study limited certain comparisons and classifications in terms of age and BMI categories of OA patients. Despite these limitations, we showed certain differences between cells obtained from BM and BMAT compartments. Some of these differences might be the consequence of different tissue processing, where adipose cells were isolated by collagenase digestion, where enzymes per se might damage surface molecules and induce intracellular changes. However, we still were able to identify certain differences within adipose tissue from distinct skeletal and body sites.

## 5. Conclusions

Our results elucidated different lipid profiles of BMAT obtained from acetabular and femoral bones in OA patients. We delineated femoral BMAT from acetabular BMAT and gfSAT in terms of triglyceride content, the FA profile, and elevated presence of hematopoietic progenitors. Although we did not provide evidence directly contributing to the understanding of OA pathology and therapy development, our findings might improve the knowledge on BMAT biology in patients undergoing hip arthroplasty. In addition, for the first time, we evaluated human BMAT-related hematopoietic cell populations, suggesting the importance of hematopoietic cell response to stress (such as inflammation/infection) that occurred in OA patients.

## Figures and Tables

**Table 1:** The most abundant (>1%) presence of fatty acids in human adipose tissue samples.

Abbreviation	Acid	IUPAC Name
14:00	Myristic	tetradecanoic acid
16:00	Palmitic	hexadecanoic acid
16:1n-7	Palmitoleic	(Z)-hexadec-9-enoic acid
18:00	Stearic acid	octadecanoic acid
18:1n-9	Oleic acid	(Z)-octadec-9-enoic acid
18:1n-7	cis-Vaccenic	cis-11-octadecenoic acid
18:2	Linoleic	12Z-octadecadienoic acid

## Data Availability

All data generated during this study are included in this published article and its Supplementary Information Files.

## Supplementary Materials

The following supporting information can be downloaded at: https://www.mdpi.com/article/10.3390/metabo15010016/s1.
