# Supplementary material for: Skeletal Site-Specific Lipid Profile and Hematopoietic Progenitors of Bone Marrow Adipose Tissue in Patients Undergoing Primary Hip Arthroplasty
Source: Metabolites. 2025 Jan 4;15(1):16. doi: 10.3390/metabo15010016 (PMC11767117; doi:10.3390/metabo15010016)
Supplement: Supplementary file 1 [file metabolites-15-00016-s001.zip › metabolites-3392448-supplementary.pdf]

# Supplementary Materials

# Skeletal Site-Specific Lipid Profile and Hematopoietic Progenitors of Bone Marrow Adipose Tissue in Patients Undergoing Primary Hip Arthroplasty

**Drenka Trivanović [1,*], Marko Vujačić [2,3,†], Aleksandra Arsić [4,†], Tamara Kukolj [1], Milica Rajković [1], Nikola Bogosavljević [2,3], Zoran Baščarević [2,3], Mirjana Maljković Ružičić [5], Jovana Kovačević [5,6] and Aleksandra Jauković [1]**

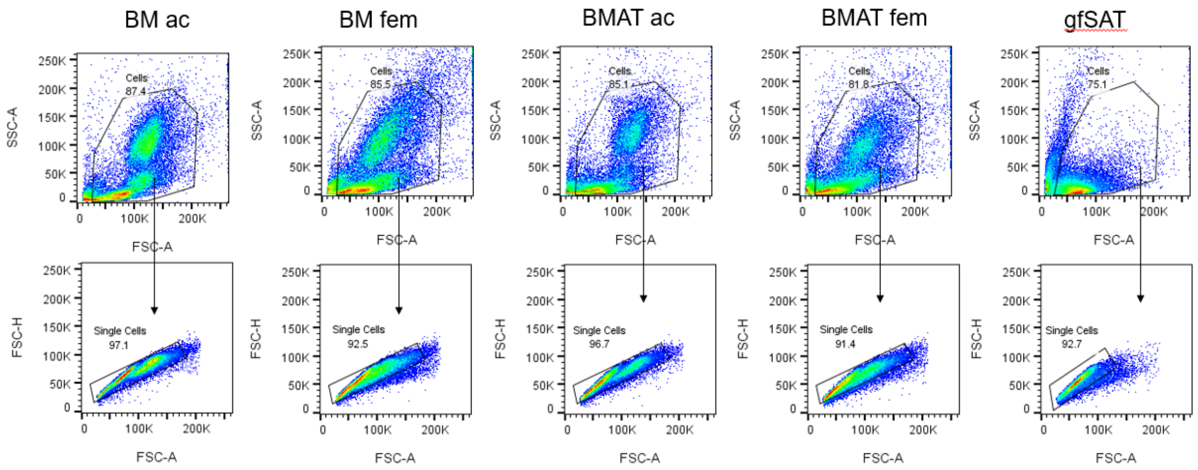


Gating strategy applied for analyses of cells isolated from different skeletal and adipose tissue compartments.

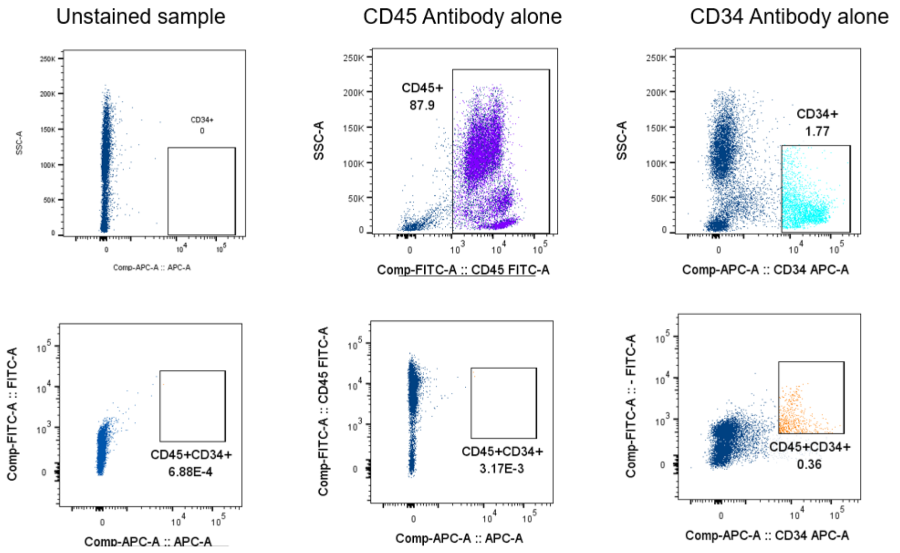


Gating strategy and control staining for analyses of CD34+ cells within different skeletal and adipose tissue compartments.
